# Tremendous and infrequently adenoid cystic carcinoma of the breast without any metastasis for more than 20 years: A case report

**DOI:** 10.1097/MD.0000000000039461

**Published:** 2024-08-23

**Authors:** Ye Liu, Lichao Zhu, Lei Guo, Haiyuan Yang, Shuqing Wang, Guoxin Sun, Jiang Li, Kuan Liu, Changyou Wang, Shengli Huang, Yating Zhao

**Affiliations:** a Breast Disease Treatment Center, Affiliated Hospital of North China University of Science and Technology, Tangshan, Hebei, China; b Gastrointestinal Diagnosis and Treatment Center, Affiliated Hospital of North China University of Science and Technology, Tangshan, Hebei, China; c North China University of Science and Technology, Tangshan, Hebei, China; d Tangshan Maternal and Child Healthcare Hospital, Tangshan, Hebei, China; e Department of Hepatobiliary and Pancreatic Surgery, Affiliated Hospital of North China University of Science and Technology, Tangshan, Hebei, China.

**Keywords:** no metastasis, rare breast cancer, tremendous adenoid cystic carcinoma of the breast, tremendous tumor, triple-negative breast cancer

## Abstract

**Rationale:**

Breast adenoid cystic carcinoma is an extremely rare tumor that is incompletely understood, accounting for less than <0.1% of all breast cancers, with an average diameter of 3 cm, and it is extremely rare to see a large, non-metastatic breast adenoid cystic carcinoma with a diameter of about 30 cm. Since this disease is extremely rare, there are few reports in the literature and limited data on clinical diagnosis and treatment. We present a case of a 71-year-old woman with a large, non-metastatic adenoid cystic carcinoma of the left breast and share our opinion on the diagnosis and treatment of this case.

**Patient concerns:**

A 71-year-old woman with a 20-year-old left breast mass with local bleeding and rupture for 1 hour presented to our hospital for further diagnosis and treatment. A computed tomography scan showed a large soft tissue mass shadow in the left breast and malignancy was considered. Subsequently, tissue aspiration pathology was performed and the results confirmed adenoid cystic carcinoma of the breast.

**Diagnosis:**

Intraoperative pathology results of radical mastectomy for left breast cancer diagnosed adenoid cystic carcinoma of the breast and immunohistochemistry results of triple-negative breast cancer.

**Interventions and Outcomes:**

Treatment of adenoid cystic carcinoma of the breast included neoadjuvant chemotherapy for breast cancer, radical mastectomy of the left breast, and postoperative chemotherapy. Initially, neoadjuvant chemotherapy for breast cancer was performed, and the TAC regimen was used to successfully reduce the size of the tumor and gain access to surgical treatment for breast cancer. The patient has recovered well after the surgery, with no wound infection or ulceration, and is now waiting for the patient’s physical function to recover for postoperative chemotherapy, with no obvious discomfort.

**Lessons:**

Adenoid cystic carcinoma tumors are usually around 3 cm; such a huge 30 cm adenoid cystic carcinoma of the breast is extremely rare, and it is extremely rare to find a breast malignancy that has not developed regional lymph node and distant metastases for more than 20 years. Clinicians must remain vigilant for early breast malignancies at a high age of incidence and conduct further research for diagnosis to avoid delays.

## 1. Introduction

Breast adenoid cystic carcinoma (BACC) accounts for approximately 0.06% to 0.1% of all breast tumors ^[1]^ and is a unique and rare specific type of breast malignancy. Adenoid cystic carcinoma is most common in the salivary glands and can also occur in the lacrimal glands, external auditory canal, upper respiratory tract and lungs, and female genital tract.^[2]^ Adenoid cystic carcinoma of the breast is a relatively low-grade malignant tumor with an average diameter of 3 cm, and there are few cases of huge adenoid cystic carcinoma of the breast with a diameter of about 30 cm. It often occurs around the areola and in the center of the breast,^[3]^ and is clinically manifested as a slow-growing, well-defined, and mobile solid mass. Adenoid cystic carcinoma has a relatively low degree of malignancy, but breast malignancies without regional lymph node and distant metastases for more than 20 years are extremely rare. Most patients are treated with surgical excision, the role of adjuvant chemotherapy is currently controversial, and hormonal therapy is usually ineffective. Due to its histological resemblance to adenoid cystic carcinoma of the salivary gland, it is often misdiagnosed as other types of carcinoma or benign lesions. Here, we report a patient with a giant adenoid cystic carcinoma with a diameter of 30 cm and no lymphatic or distant metastases for more than 20 years. The clinical diagnosis, course, and pathological features are discussed and reviewed in this article.

## 2. Case report

**Figure 1.**
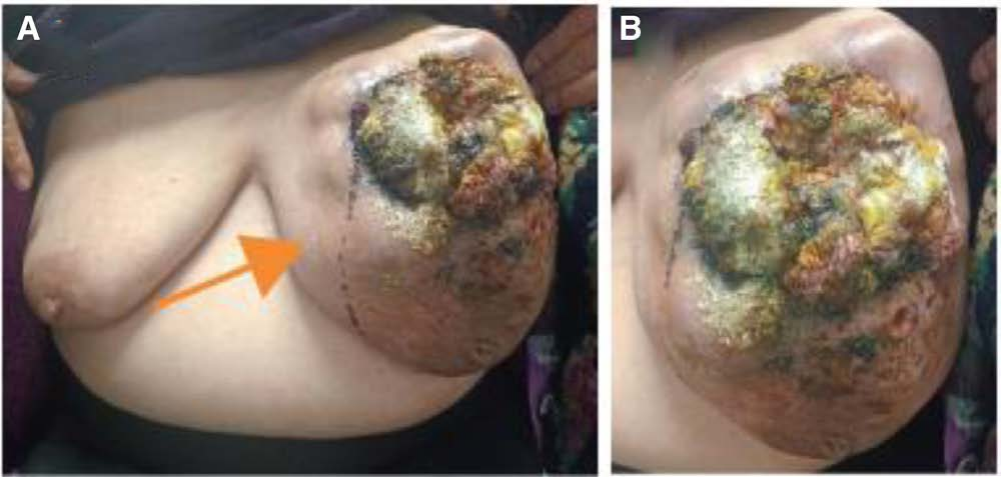
Breast manifestations at the time of the patient’s visit (A and B).

**Figure 2.**
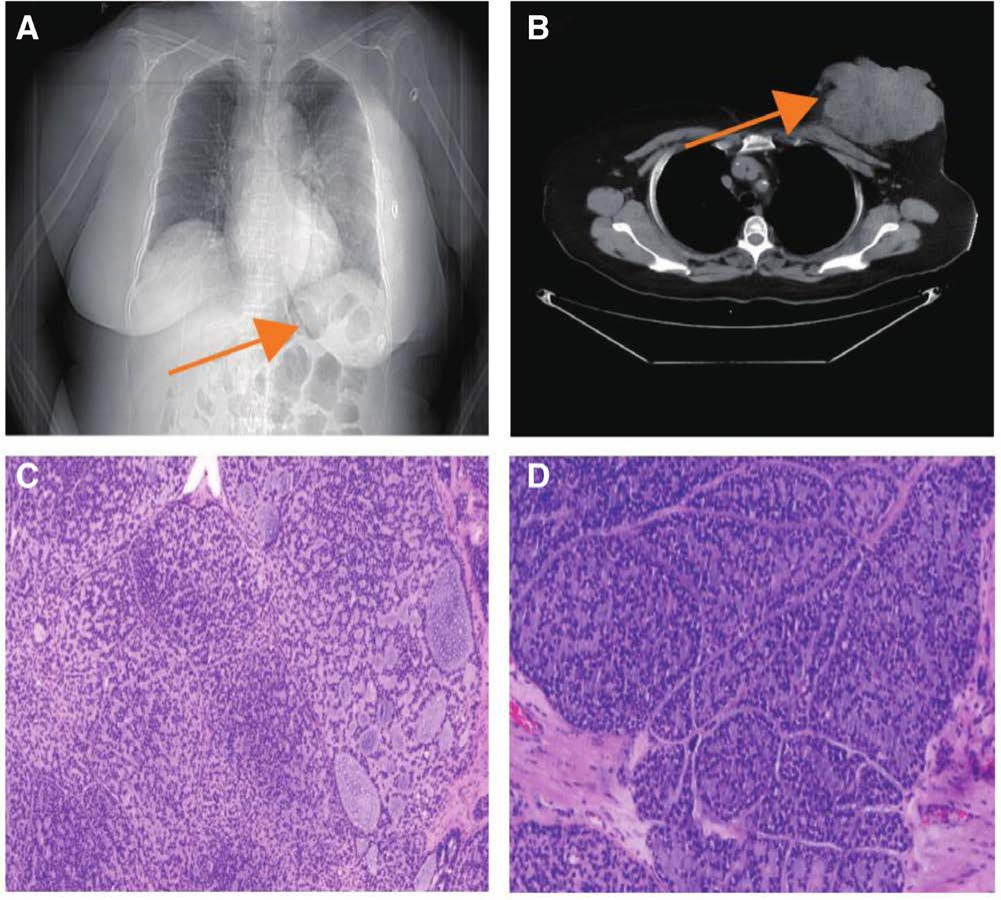
(A and B) Chest computed tomography findings: a large soft tissue mass of approximately 30 cm in diameter in the left breast. (C and D) Pathological findings of breast puncture.

A 71-year-old woman from Hebei Province, China, was admitted to our hospital on February 3, 2024 with a 20-year-old left breast lump that had been locally broken and bleeding for 1 hour. The patient found the left breast lump unintentionally 20 years ago, which was about the size of a “fava bean”, with no pain, no nipple overflow, and no skin redness or swelling. The patient did not seek medical treatment, and the swelling gradually increased in size over the past 20 years, and is now about the size of a “cantaloupe”, with a cauliflower-shaped swelling that is solid out of the skin surface, and which was locally ulcerated and bled 1 hour ago. The patient had no past medical history. Physical examination showed that the left breast was visible as a mass of about 30 × 25 cm in size protruding from the skin surface, which was hard, irregular, fixed in position, covered with a large amount of purulent secretion, accompanied by a foul odor, with local skin ulceration and hemorrhage, and the skin of the left lower quadrant of the breast was red, swollen and cellulite-like, and a number of swollen lymph nodes could be detected in the left armpit, with a maximum of 2.0 × 1.0 cm in size, which were hard and with a still clear border, and could be moved without pressure or pain. Mobility was acceptable and there was no pressure pain (Fig. 1A and B). Laboratory tests showed an elevated erythrocyte count (5.25 × 10^12^ cells/L) and neutrophil ratio (6.83 × 10^9^ cells/L). Computed tomography showed a large soft tissue mass in the left breast, which was considered to be a malignant tumor (Fig. 2A and B).

**Figure 3.**
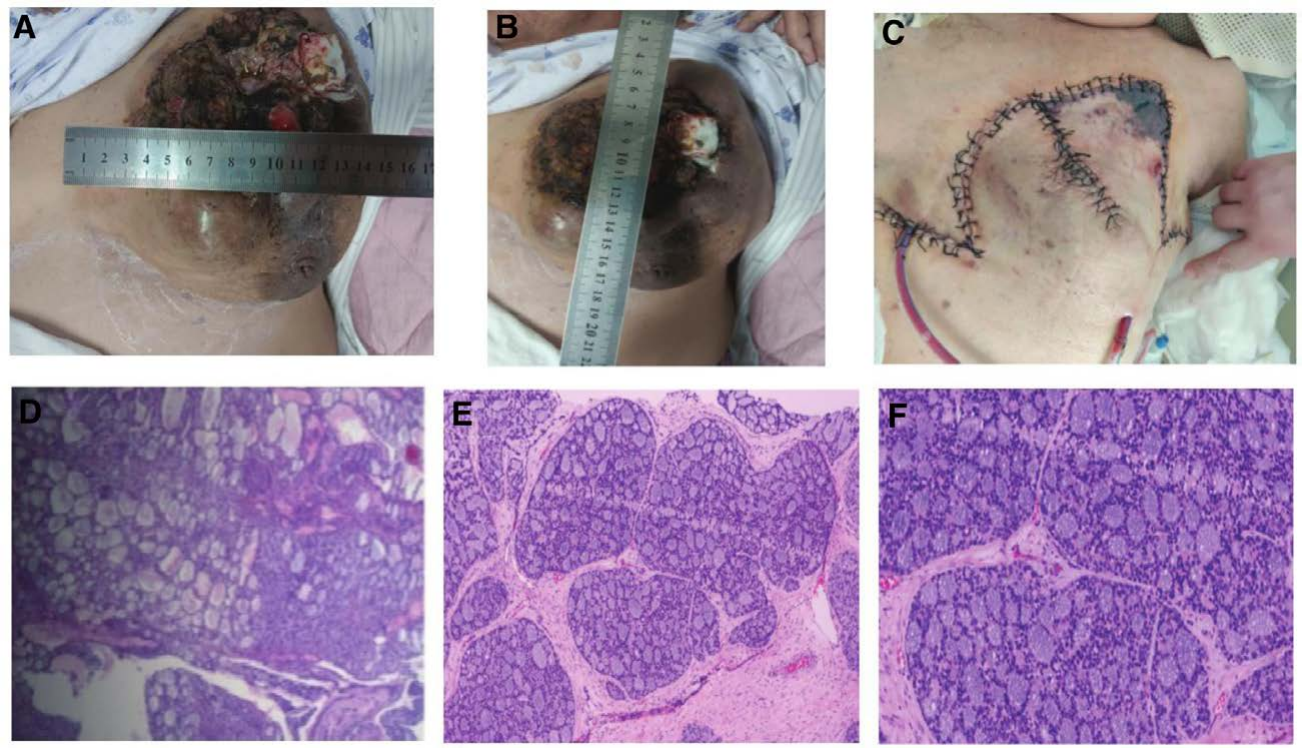
Neoadjuvant therapy for breast cancer (A and B). The first day after radical left breast cancer surgery (C). Intraoperative pathological findings of radical left breast cancer (D and F).

Combined with preoperative pathological puncture results (Fig. 2C and D) and immunohistochemical phenotyping: tumor cells were negative for estrogen receptor (ER), progesterone receptor (PR), human epidermal growth factor receptor-2, calponin, Syn, CD56, CgA, and CK5/6; positive for P120 (membrane +), E-Cadherin, p63, EGFR, BRCA1, and TRPS-1 were positive; CD117, CK8/18, PAS, AB, and CD117 were partially positive; and the Ki-67 positivity index was 50%, which led to the diagnosis of triple-negative breast cancer. According to the Chinese Society of Clinical Oncology Breast Cancer Guidelines, neoadjuvant treatment for breast cancer was performed, and the TAC regimen was adopted, with doxorubicin hydrochloride liposome injection liquid 40 mg, cyclophosphamide 800 mg, and albumin paclitaxel 400 mg on static drip. After 1 cycle of chemotherapy, 21 days later, the patient came to the hospital for follow-up, and the left breast mass was significantly reduced (Fig. 3A and B), which was in line with the indications for surgery, and the radical mastectomy of the left breast cancer + adjacent flap transfer was performed with a circumferential incision under general anesthesia.

Combined with intraoperative findings (Fig. 3C) and intraoperative pathological examination (Fig. 3D and E), the diagnosis was adenoid cystic carcinoma of the left breast cT4N1M0, classic, with no cancer involvement in the upper and lower medial and lateral margins, papillary muscle and pectoralis major and minor muscles, and no metastatic cancer in any of the lymph nodes. The first day of postoperative recovery after radical mastectomy for left breast cancer (Fig. 3F), the postoperative wound healed well without redness, swelling, rupture, and other inflammatory and adverse manifestations. At present, the patient has no obvious discomfort, waiting for the patient’s physical function recovery for postoperative chemotherapy. It is expected that the postoperative chemotherapy will be carried out by AC-T regimen.

## 3. Discussion

Breast adenoid cystic carcinoma (BACC) accounts for about 0.06% to 0.1% of all breast tumors,^[1]^ it belongs to a unique and rare specific type of breast malignant tumor, it usually occurs in postmenopausal females, with a median age of 60 years old, and is rare in males, it can occur in both breasts, it preferably develops in the infraareolar area,^[2]^ it rarely presents with bloody nipple overflow, it has a single focal lesion in most of cases, and multifocal lesions in a few of the cases, some of the cases may present with pain or tenderness. Some scholars have referred to BACC as a specific triple-negative breast cancer, but recent studies have shown that adenoid cystic carcinoma of the breast cannot be classified as a triple-negative breast cancer because it also includes a small number of hormone receptor-positive breast cancers.^[3]^

BACC is similar to salivary adenoid cystic carcinoma in terms of tissue composition, with the cancerous tissue consisting mainly of myoepithelial cells, basal-like cells and glandular epithelial cells. According to the 2019 WHO classification basis for breast tumors, adenoid cystic carcinoma of the breast is divided into 3 histological types: classic, solid-basal-like, and high-grade transformed, and the case we report belongs to one of the more common types: classic. The structural pattern of tumor cell arrangement in BACC. There are generally 3 different types: tubular, sieve, and solid (basal-like). Sieve and solid arrangements are more predominant, while tubular is less common. Sieve and solid structures correlate with tumor aggressiveness, whereas tubular structures represent a relatively high degree of differentiation. Sequeiros et al reported that patients whose tumors showed solid-type structures had the worst prognosis, with sieve intermediate and tubular best.^[4]^

The differential diagnosis of BACC depends primarily on its structural pattern. When the sieve-like structure is predominant, it needs to be differentiated from the following diseases: (1) sieve ductal carcinoma in situ: it is an intraductal lesion, the tumor cells proliferate in the lumen of the gland in a sieve-like shape and diffusely and strongly positively express ER, PR, and do not express CD117, and the duct is surrounded by myoepithelial cells with myoepithelial markers positive; (2) invasive saphenoid or tubular carcinoma: the tumor cells consist of a single adenoepithelial cell arranged in a saphenoid/tubular pattern, with infiltrative growth, lack of myoepithelial cells, positive for ER and PR, and negative for CD117 and myoepithelial markers. (3) Collagen vesiculopathy: usually irregularly shaped collagen spherules are seen around the lesion, with no mucus-like material in the cavity, no infiltrative growth, and no expression of CD117. When tubular structures predominate, they need to be differentiated from adenomyoepitheliomas. When solid (basal-like) structures predominate, they need to be differentiated from small cell carcinoma, solid papillary carcinoma, anaplastic carcinoma, and malignant lymphoma.^[5]^

Patients with BACC have a good prognosis, with 5-, 10-, and 15-year survival rates of 98%, 95%, and 91%, respectively, lymph node metastasis rate of <8%, distant metastasis rate of <20%, and almost no axillary lymph node metastasis when the mass is <1.4 cm.^[6]^ Some studies have shown ^[6]^ that the rate of axillary lymph node metastasis in adenoid cystic carcinoma of the breast is low, and only 5% of patients with BACC had lymph node involvement. Even in other studies,^[7]^ there were no cases where axillary lymph node metastasis was detected by sentinel lymph node biopsy or axillary lymph node dissection. BACC recommends breast conserving surgery/simple mastectomy and/or sentinel lymph node biopsy and axillary lymph node dissection is not necessary if the sentinel lymph nodes are negative.^[8]^ In the case we report, despite the patient’s tumor surface breaking down and bleeding and the cancer invading the skin, the pathological findings showed no axillary lymph node metastasis, so axillary lymph node clearance was not necessary.

The diagnosis of adenoid cystic carcinoma of the breast is undoubtedly a major challenge for pathologists. For example, it is difficult to distinguish between the 4 pathological images of ductal carcinoma in situ, invasive ethmoid carcinoma, collagen glomerulopathy, and malignant adenomyoepithelioma, and it is difficult for pathologists to make accurate diagnosis. The tumor cells in ductal carcinoma in situ are composed of a single glandular epithelial cell with a polar distribution characteristic of vertical lumen. Lack of infiltrative features and presence of periductal myoepithelium. Immunohistochemical ER and PR were diffuse strong positive. Invasive ethmoid carcinoma is an invasive growth, the sieve is lined with vertical glandular epithelium, and the immunohistochemistry is often strongly positive for ER and PR, and negative for myoepithelial markers. Collagen glomerulopathy is usually a small benign lesion found by chance, showing a noninvasive growth pattern. Eosinophilic and basophilic glomerulopathy rich in collagen, laminin, and mucopolysaccharide are often clustered in the lumen, surrounded by proliferative myoepithelial cells. Both collagen glomerular disease and BACC expressed SMA and p63, but SM-MHC positive and CD117 negative in collagen glomerular disease. The BACC is the opposite.^[9]^ Malignant adenomyoepithelioma presents as adenomyoepithelioma based on malignant glandular or myoepithelial degeneration, which still retains its background as adenomyoepithelioma.

Studies have confirmed that adjuvant chemotherapy does not improve the clinical outcome of BACC in patients receiving adjuvant chemotherapy. This is true even in patients with a high risk of recurrence and metastasis, ^[7]^ whereas the role of adjuvant chemotherapy is currently controversial and hormonal therapy is usually ineffective. Early detection of BACC is challenging due to its extremely low incidence and the particular rarity of giant adenoid cystic carcinoma of the breast. Therefore, it is clinically important to have a good understanding of the clinical manifestations of the disease in order for surgeons to develop the best surgical plan and avoid overtreatment.

## Acknowledgments

We thank the patient, who agreed to the publication of her images and clinical information. Informed consent was obtained from the patient for publication of the case.

## Author contributions

**Data curation:** Haiyuan Yang.

**Funding acquisition:** Yating Zhao.

**Investigation:** Jiang Li.

**Methodology:** Ye Liu.

**Project administration:** Lichao Zhu.

**Resources:** Lei Guo, Shuqing Wang, Guoxin Sun.

**Software:** Shengli Huang.

**Validation:** Kuan Liu, Changyou Wang.

**Writing – original draft:** Ye Liu.

**Writing – review & editing:** Yating Zhao.
